# Determinants of farm mechanization in central and southeast oromia region, Ethiopia

**DOI:** 10.1016/j.heliyon.2023.e18390

**Published:** 2023-07-17

**Authors:** Tamrat Gebiso, Mengistu Ketema, Arega Shumetie, Getachew Leggesse

**Affiliations:** a Candidate at Haramaya University and Oromia Agricultural Research Institute, Ethiopia; b Professor of Agricultural Economics and CEO of Ethiopian Economics Association, Ethiopia; c Senior Researcher at Ethiopian Economics Association, Ethiopia; d Senior Researcher at International Livestock Research Institute (ILRI), Ethiopia

**Keywords:** Farm mechanization level, Tobit, Mechanization index, Tractors, Combine harvester

## Abstract

The study has been conducted to assess the farm mechanization level and tried to identify the determinants. The research utilizes primary data, collected through personal interview of 397 farm households located in the four districts of central and southeast Oromia region of Ethiopia. Multi-stage, purposive and random sampling procedures were used to select the respondents using probability proportional to size from each district. Mechanization Index (MI) based on the matrix use of animate and mechanical energy inputs that incorporate cost factors was used to estimate farm mechanization level, while Tobit model was employed to analyze factors that determine the farm mechanization level. Household’s sex, educational background, experience in farming, family labor availability and social capital, location of household, access to all-weather roads and distance to farm mechanization service providers centers, participation in market and off-farm activities, landholding, land fragmentation and size of livestock owned (TLU) are statistically significant in determining level of farm mechanization. Land consolidation, availing infrastructural facilities and facilitating adult education and short-term trainings are important recommendations to enhance farm mechanization level in the study area.

## 1 Introduction

Low productivity of sub-Saharan African (SSA) agriculture is attributed to low application of science and technologies. Despite African leaders’ Maputo Declaration to spend at least 10% of their national budget to support agriculture [1], an important input to transform and modernize the sector, farm mechanization, was neglected in the region for a long time [2,3]. However, recently farm mechanization demand in Africa as a whole and in SSA in particular is increasing and there are policy changes towards promoting it [1,4]. Increase in farm labor shortage, the rise in wages in the agricultural sector due to out-migration of labor, and the growing need of sustainable intensification practices to increase food production and the input use efficiency in the sector are the main driving factors to farm mechanization in developing countries like Ethiopia [5].

The Ethiopian agricultural sector continued to grow in both growth and transformation plan (GTP I and GTP II) between 2011 and 2020. It showed an average growth rate of 5.3% for the last ten years and contributed a significant share of the national GDP that is about 33%. The well above national population growth rate of the sector contributed in reducing the poverty level from 30.4% to 25.6% between 2015 and 2020 [6,7]. It plays an important role in the national economy by absorbing the majority of the labor force (70%) and generating the highest share of foreign exchange *i.e*., 68% of export value [6]. This considerable and continuous contribution of the sector was attributed to factors such as use of modern farm inputs, rapid expansion of cropland, increased labor productivity, and government’s investment in extension system and an improved road network [8], which is mainly due to intensification and area expansion similar to that of other SSA countries [5]. However, there are also efforts to continue sustainable farm mechanization [9].

Level of farm mechanization in Ethiopia is among the lowest in Sub-Saharan African (SSA) countries. The study shows that out of nine SSA countries, which are Burkina Faso, Ethiopia, Ghana, Kenya, Mozambique, Nigeria, Rwanda, Tanzania, and Zambia, Ethiopia is the second from last by having only 4 tractors per 100km2 of lands topping only Rwanda. And the result was by far below SSA average which was 13 tractors per 100km2 [10]. However, the annual report by the Ethiopian Ministry of Agriculture (MoA, 2023) shows that currently there is an improvement of large on per km2 coverage of the tractors at national level following national policy reforms. According to the data, there are 14,968 tractors, 4486 combine harvesters, 8000 motorized thresher, and 157414 irrigation pumps in Ethiopia. Based on this annual report (Annex 1) and CSA [11] data of total cultivated land, the per 100km2 tractor coverage is increased to 10.57 tractors where the size of tractors under use ranges from 20 to 130 horse power. Wheat, maize, teff and sorghum are relatively more mechanized crops according to their order in Ethiopia [12].

The spatial patterns of farm mechanization in Ethiopia shows that there is relatively large concentration in the Oromia region specifically the Arsi-Bale area, Western Tigray and parts of Somali region. The concentration to these areas is obviously attributed to historical intervention, the presence of commercial and state farms, relatively larger farm holdings for smallholders, higher rural wages, suitability of land for large mechanization, and the bimodal nature of rain which enables farmers to practice double cropping and that add pressure on farmers, hence necessitated the use of farm machinery [un-published report of DBE, 2018 & MoA, 2023).

The rise in rural wages and cost of animal traction were the most common factors forcing farmers to use farm mechanization in Ethiopia, among others. The continuous rural wage increment was due to children’s schooling and massive migration to urban and outside the country [13]. Rural wages of unskilled labor grown by 54% whereas, the same grown by 63% in urban areas of Ethiopia between the years 2004 and 2015, whereas the gap grown by 5.5% between urban and rural wages [13]. Moreover, cost of animal traction is fueled due to increases in cost of maintaining traction animals due to scarce grazing land.

Even though the use of tractors for tillage and engine-driven threshers and combine harvester were common in central highlands of Arsi and Bale since the 1950’s mainly as a result of policies favoring large scale farms to import duty free machineries and parts and subsidized fuels [14,15], it overlooked small scale farms and caused eviction of tenants and end-up with conflicts and stagnation of mechanization processes in the country. The military socialist government of Dergue overtaken all large private farms of imperial period and transferred them to state farms, and tried to maintain their level of mechanization but private farm mechanization was not encouraged [16,17].

After the overthrow of the *Dergue* regime, small scale farms in particular and agriculture sector as a whole has been given better policy attention [17,18], that result in providing focus for small scale farm mechanization. Moreover, the federal government of Ethiopia in general and the regional government of Oromia in particular, have given especial attention to farm mechanizations recently where duty free imports, machinery loans from the development bank and other private banks like Cooperative bank of Oromia many other incentives were provided to farmers. However, unlike other agriculture related issues in Ethiopia, studies on farm mechanization are very limited and there are no quantified research outputs on level of farm mechanization, socioeconomic, demographic and other variables that affect the up-take of farm mechanization in Ethiopia in general and Oromia region particularly. Hence, the research was initiated with the objectives of (i) estimating the level of farm mechanization and (ii) identifying determinants of the farm mechanization level in the study area.

## 2 Methodology of the study

### 2.1 Description of the study area

Oromia is the largest national regional state in Ethiopia both in terms of population and land size by having 35.1% and 32% of total national population and land area, respectively [19]. Cereals are the most important crops in the region, which covers around 84% of total grain crop land. *Teff*, maize, wheat and sorghum are the most common cereals in terms of area coverage and volume of production. From vegetables, red peppers, Ethiopian cabbages and green peppers are widely grown while root crops like potatoes, sweet potatoes and onions are among the majors in terms of area coverage [11].

Arsi and West Arsi zones are found in central and southern part of Oromia national regional state, respectively. Geographically, Arsi zone lies between 6^0^ 45ʹN to 8^0^ 58’N latitude and 38^0^ 32’E to 40^0^50ʹE longitudes while West Arsi zone lies between 6^0^12ʹ29ʹ to 7^0^42ʹʹ55ʹ latitude and 38^0^4ʹʹ04ʹ to 39^0^46ʹʹ08ʹ longitude. Because of its diversity in altitude, Arsi zone has high physiographic diversity. Based on the altitude, there are four major identified physiographic divisions, which are cool; cool temperate; warm temperate and lowlands agroclimatic zone [20]. Similarly, West Arsi zone also has three major agroclimatic zones - highland, midland and lowland. These two zones, the study areas, have complex and interlinked and diversified mixed crop-livestock farming systems. For instance Ref. [20], broadly categorized farming typologies of Arsi into seven. However, there is a dynamism of farming system in the area and following the current government policy change in the sector, introduction of mechanization is becoming common.

### 2.2 Sampling design and sample size determination

The study considered households as a decision-making unit and considered farm household heads as key respondents and decision points of the household’s farm and non-farm activities. A multi-stage sampling procedure was employed to draw the target sample households. First, two zones, Arsi and West Arsi were selected purposively based on representativeness to the area for study objectives. Highland and mid-highland districts that have good access to farm mechanization were selected based on secondary data and discussions held at the zonal office of agriculture. At the second stage, four districts, Kofele and Gedeb Asasa from West Arsi and Lemu-Bilibilo and Hetosa from Arsi were selected randomly. Kofele and Lemu-Bilbilo are representing highland while Hetosa and Gedeb-Hasasa were from mid-highland districts. At the third stage, the random sampling procedure was employed to draw two *kebeles* from each district and accordingly a total of eight *kebeles* were selected. Finally, the total sample size was determined using a formula, which provides a representative size to ensure the desired precision using the formula given by Ref. [21]:

1 $$N=\frac{Z^{2}pq}{e^{2}}=\frac{{\left(1.96\right)}^{2}\left(0.5\right)\left(0.5\right)}{{\left(0.05\right)}^{2}}=385$$

Where, N is the desired sample size; Z is the standard cumulative distribution that corresponds to the level of confidence with the value of 1.96; e is desired level of precision; p is the estimated proportion of an attribute present in the population with the value of 0.5 as suggested by Ref. [22] to get the desired minimum sample size of households at 95% confidence level and ±5% precision; q = 1-p. Accordingly, a sample of 397 household’s heads was selected and interviewed using random sampling with probability proportional to size.

### 2.3 Data sources, types and methods of data collection

This study used data from both primary and secondary sources. Primary data sources were mainly farming households and mechanization service renders (cooperatives or private machinery owners). Secondary data were collected from published and unpublished sources like CSA, official reports, government policy documents and journals. Primary data were collected through face-to-face personal interviews using structured questionnaires. The questionnaires were developed based on previous research outputs on determinants of farm mechanization adoption in Ethiopia and other countries [[23], [24], [25], [26], [27], [28], [29], [30], [31], [32], [33], [34], [35], [36], [37], [38], [39]]. Mixed type of research method was employed in this study as the sole approach of research method, either qualitative or quantitative, may not be enough to grasp the major constraints in agricultural mechanization. Hence, it is recommended to supplement quantitative methods by qualitative methods [40]. A mixed methods of research is a combination of techniques which is defined as an intellectual and practical synthesis based on qualitative and quantitative research [41] and involves combining these two strands and data in response to respective research questions [42]. Accordingly, FGDs (one per *kebele)* and Key Informant Interviews (KII) were conducted to enrich and triangulate the data from the questionnaires. An average of 8–14 discussants were selected at each kebele by considering sex, educational background and age to conduct FGD. KII was conducted with development agents at each kebele, experts from zonal and district office of agriculture and union managers. The field surveys were conducted by trained staffs from Asella agricultural research center using The Census and Survey Processing System (CSPro) software.

### 2.4 Methods of data analysis

Both descriptive and econometric models were used in this study. The descriptive statistics like mean, tabulation and index computation were made to summarize socioeconomic characteristics of the households, types of farm mechanization, constraints in farm mechanization and to the estimate level of farm mechanization. The study employed Tobit model to analyze farm mechanization status and its determinants.

### 2.5 Estimating the level of farm mechanization

Level of farm mechanization is determined by using Mechanization Index (MI). Following [43], MI based on the matrix use of animate and mechanical energy inputs, it could be given by incorporating cost factors:

(2) $$MI=\frac{C_{Mi}}{C_{Hi}+C_{Ai}+C_{Mi}}\;)\;*\;100$$

Where, MI is mechanization index expressed in percentage; C_Mi_, C_Hi_, and C_Ai_ are costs of using machinery, human labor and animal power by ith household per hectare, respectively for wheat and barley crop production. This method is preferred due to data type we can collect from farmers on a recall basis because of absence of farm records. Hence, energy-based index like kilojoules per hectare (KJ/ha) methods are not preferred in this study.

Determinants of farm mechanization level: Specification of Tobit model:

Level of mechanization is an index that lies between zero (non-mechanized) and 1 (completely mechanized). Therefore, to identify factors that determine the level of farm mechanization, ordinary least square (OLS) could be biased and inconsistent [44], and hence instead, a censored regression model developed by Ref. [45] called Tobit, is proposed in this study. Therefore, following [46], the Tobit model can be specified as in equation (3):

(3) $$Y_{i}^{*}=\beta^{\prime }X_{jk}+u_{i}$$

If Y_i_ is denoted as the observed dependent (censored) variable, then it can be given as equation (4):

(4) $$Y_{i}=\left\{\begin{array}{c} Y_{i}^{*}=L\;ifY_{i}^{*}\leq L\\ Y_{i}^{*}=L\;if\;L<Y^{*}<U\\ Y_{i}^{*}=U\;ifY^{*}\geq U \end{array}\right\}$$

Where: *Y*_*i*_ is the observed dependent variable, in this case farm mechanization level of household i (unobserved for values below 0 and above 1), *X*_*jk*_ is a vector of explanatory variables for household k (l = 1, 2, .., j) and *u*_*i*_ an error term that is iid with mean zero and variance $\delta^{2}$, and assumed to be independent of X_jk_. The distribution of the dependent variable in equation (3) is not normal since its value varies between 0 and 1. The likelihood function of this model, following [46], is specified as in equation (5):

(5) L(β,δ/yjXjL1jL2j)=Πyj−L1jφ(L1j−β*Xjδ)Πyj=yj*.φ(yi−β*Xjδ)Πφyj−L2j(L2j−β*Xjδ)

Where: L_1j_ = 0 (lower limit) and L_2j_ = 1 (upper limit) are normal and standard density functions.

## 3 Results and discussion

### 3.1 Descriptive analysis of variables in tobit model for farm mechanization level

Table 1 below presents a summary of explanatory variables included in the Tobit model. It shows that 96% of the household heads are male-headed. Mean farming experience, educational level and family labor of the sample households are around 23 years, 6 grades and 2man equivalent, respectively, while mean cultivated land size and livestock holding are 5.18 TLU and 1.52 ha. Average distance from mechanization service providing centers and market places is 6.24 kms and 6.78 kms, respectively. Around 88% of the sample households have access to all-weather roads. Mean crop diversification index in the Simpson diversification index (SDI) is 0.47 while each household has 2.23 plots on average.

**Table 1:** Descriptive analysis of variables in Tobit model.

Variable	Mean	Std. dev.	Min	Max
Household head sex (1 = male; 0 = female)	0.96	0.197	0	1
Education of head (grade)	5.94	4.047	0	15
Farm experience (year)	22.60	12.921	1	70
Location (1 = W/Arsi; 0 = Arsi)	0.51	0.500	0	1
Mech. Center distance (Kms)	6.42	5.263	0.05	45
Cultivated land (ha)	1.52	1.164	0.1	7
Number of plots (counts)	2.23	1.131	1	6
Family labor (man-equivalent)	2.34	1.599	1	12.6
Market participation 1 = yes; 0 = No)	0.81	0.394	0	1
Off farm activities 1 = yes; 0 = No)	0.17	0.377	0	1
Livestock owned (TLU)	5.18	4.135	0	34.05
Access road (1 = yes; 0 = No)	0.88	0.326	0	1
Market distance (Kms)	6.78	4.881	0.1	25
Crop diversifying (SDI)	0.47	0.257	0	1
Social capital (Index)	0.47	0.201	0	1

### 3.2 Asset ownership by sex of household head in the study area

Table 2 below shows the asset and family labor in man equivalent availability of households by sex of the household’s head. The result shows that male headed households have a greater number of oxen and more size of livestock in TLU. Similarly, landholding and family labor are more for male headed households than female headed households. Level of farm mechanization in MI is also higher for female but statistically insignificant where it is 0.35% and 0.46% for male and female headed households respectively. The higher in MI for female headed households could be due to less family labor and number of oxen possessed by female headed households which are substitutes for farm mechanization.

**Table 2:** Asset ownership and labor availability variables across sex (gender) of household head.

Variables	Mean			
	Male	Female	Pooled mean	t-value
Number of oxen	1.43	1.11	1.43	0.801
Livestock (TLU)	5.14	5.46	5.15	0.241
Family labor (man equivalent)	2.33	1.94	2.32	0.722
Total landholding (ha)	1.93	1.79	1.93	0.325
Educational status (grade)	6.08	3.00	6.01	2.284
Mechanization Index (MI)	0.35	0.46	0.35	1.408

### 3.3 Types and level of farm mechanization technologies

Respondent households are using different farm mechanization technologies, whereas agricultural farm implements that are operated by animal power are owned by almost all of the households. The widely used modern farm mechanization technologies are four-wheel tractors and combine harvesters while there are two-wheel tractors and engine-driven stationary threshers adopted by a few households. The majority of the households adopt either of four-wheel tractors (86.15%) and combine harvesters (77.83%) or both. The result further shows that 86.66% of the households are using any of inanimate agricultural mechanization technologies. According to the official report from the districts and zonal offices of agriculture, the majority of the households in the study areas are using farm mechanization (Table 3). The result further shows that more than 70% of the households use combine harvesters and tractors or either of the two technologies. The most common sources of the farm machineries are mainly private owners and unions like Galema and Hetosa. Oromia seed enterprise and Ardayta ATVET college also provide rental services for neighboring smallholder farmers during their slug time. The power of tractors operating in Ethiopia as a whole are ranging from 20 to 130 HPs (MoA, 2023).

**Table 3:** Types and main reasons behind adopting farm mechanization technologies.

Description of variables	Frequency	Percent
Farm mechanization type used by households:		
Two-wheel tractor	80	20.15
Four-wheel tractor	342	86.15
Engine-driven threshers	55	13.85
Combine harvester	309	77.83
Used either of mechanization technologies	348	87.66
Reasons to look at mechanization options:		
Labor shortage	200	50.38
High wage rate for hired agricultural labourer	197	62.97
High animal feed cost	254	63.98
To reduce work drudgery (better life)	145	36.52

Four main reasons have been identified behind adopting farm machineries. Accordingly, high cost of keeping agricultural working animals both for land tillage and threshing purposes is selected as the main problem by around 64% of respondents, while the agricultural labor shortage was the second main reason wherein 53.38% of the respondents ranked as the main driving factor towards farm mechanization. Moreover, family and hired labor shortage is becoming a critical problem due to schooling and migration of youths into urban areas in search of better life. Hired labor is neither available nor affordable due to high wage rate. The increase in wage may be due to an increase in the educational status of the society and hence they are either not willing to do such drudgery works or demanding better payments. Farming community’s need for better (non-drudgery) life and modernization of agriculture was the other main driving factor of farm mechanization, and significant respondents i.e., around 37% identified this factor as a driver of agricultural mechanization.

Level of farm mechanization is computed for the two major crops namely wheat and barley which covers around 81% of total land in the study area. Table 4 shows that average land size allocated for barley and wheat crops is 1.39ha, from the average landholding of the household that is 1.88ha. The average wheat and barley farm land allocation of the households is 0.86ha and 0.53ha, respectively. Level of farm mechanization (Mechanization Index-MI) was calculated by using equation (1). According to the result, wheat MI ranges from zero to 100% while its mean value is 38.46%, which implies that around 39% of the wheat production operation are performed by machineries such as tractors and combine harvesters. In line with this, mean barley MI is 34.89% while the compound MI for the two crops is 35.13%. Hence, the result reveals that wheat farming is the more mechanized practice in the area.

**Table 4:** Land allocation and level of mechanization for wheat and barley production.

Land coverage	Mean value	St. Deviation	Min	Max
Total land (ha)	1.88	1.30	0.02	10
wheat farm size (ha)	0.86	1.69	0	10
Barley farm (ha)	0.53	0.59	0	4
Ratio of major crop to total land	0.81	0.84	0	10
Major crop farm size (ha)	1.39	1.75	0	10
Mechanization Index for major crop				
Variable	Mean value (%)	St. Deviation	Min	Max
MI_Wheat	38.46	0.25	0	100%
MI_Barley	34.89	0.31	0	100%
MI for Major crops (wheat and barley)	35.14	23.17	0	100%

### 3.4 Major constraints in mechanized agriculture in the study area

Table 5 presents the constraints hindering sample households from using farm mechanization and their levels of severity. Accordingly, 73.55% and 46.22% of respondents indicated that high price and access to finance are the main constraints for farm mechanization respectively. In addition to these, unavailability of the technology is the next problem to use farm machineries. One can also derive that the topography of the farm land is convenient to use farm mechanization technologies wherein more than 72% of the respondents mentioned that the topography is not a problem at all or very low problem. According to the result from FGD, inconvenient topography and technology accessibility in terms of availability are among the most important constraints in the highland areas, which are Kofele and Lemu-bilbilo districts. The households’ response also showed that most constraints are ranked as “medium” level.

**Table 5:** Major constraints in using farm mechanization technologies and their severity.

Constraints	Severity of the constraints					
(Variables)	Highest		Medium		Low	
	“Yes” Response	“Yes” %	“Yes” Response	%	Response	%
Technology Unavailable	90	22.67	147	37	86	21.66
High price	292	73.55	71	17.88	26	2.55
Technical Incapability	60	15.11	155	38.04	147	37.03
Ignorance	43	10.83	129	32.49	163	41.06
Topography	32	8.10	78	19.75	93	23.76
Poor extension	37	10.00	199	53.78	99	26.76
Finance problem	171	46.22	106	28.65	64	17.30

### 3.5 Determinants of farm mechanization level: tobit model output

A total of 15 explanatory variables is included in the model. The likelihood function of the model for the level of the farm mechanization index is highly significant (F (15, 382) = 7.43; Probe > F = 0.0000), which evidenced a strong explanatory power of the independent variables. A total of twelve socioeconomic and demographic regressor variables is significant in determining the level of the farm mechanization index (Table 6).

**Table 6:** Tobit estimate and marginal effects of determinants of level of farm mechanization.

Variable	$\frac{\partial E\left(y*|x\right)}{\partial x}=\beta$	Std. Err.	P > t	$\frac{\partial E\left(y|x\right)}{\partial x}=\beta \Phi \left(\frac{x\beta }{\sigma }\right)$	$\frac{\partial \mathrm{Pr}\left(y>0|x\right)}{\partial x}=\varphi \left(\frac{x\beta }{\sigma }\right)\frac{\beta }{\sigma }$
Sex of head (Male = 1)	−0.1059	0.0081	−3.98***	−0.0920	−0.0322
Farm experience (years)	0.0026	0.0005	2.39**	0.0022	0.0013
Education of head (grade)	0.0066	0.0015	2.23**	0.0056	0.0033
Location (W/Arsi = 1)	−0.0498	0.0134	−1.85*	−0.0419	−0.0247
Off farm activities (yes = 1)	0.0438	0.0101	1.89*	0.0373	0.0190
Cultivated landholding (ha)	0.0270	0.0058	2.32**	0.0227	0.0134
Livestock (TLU)	−0.0080	0.0016	−2.46**	−0.0067	−0.0039
Number of plots (count)	−0.0501	0.0060	−4.18***	−0.0422	−0.0249
Family labor (man-equiv.)	−0.0058	0.0035	−0.83	−0.0049	−0.0029
Market participation (yes = 1)	0.0911	0.0230	2.58***	0.0747	0.0593
Access to road (yes = 1)	0.0707	0.0222	2.04**	0.0580	0.0452
Market distance (kms)	0.0009	0.0012	0.38	0.0008	0.0005
Mech. center distance (km)	−0.0094	0.0013	−3.71***	−0.0079	−0.0047
Crop diversification (SDI)	0.0470	0.0262	0.89	0.0396	0.0234
Social capital (Index)	−0.1307	0.0280	−2.32**	−0.1099	−0.0649
Constant		0.0802	5.67***		
Number of observations = 397; Uncensored = 381					
Left-censored observations = 11; Right-censored observations = 5; y = Pr (0 < MI < 1) = 0.9486					
Log pseudolikelihood = 30.3159; Pseudo R^2^ = 2.6158; y = E (MI|0 < MI < 1) = 0.3709					
F (15, 382) = 7.43; Prob > F = 0.0000; y = E (MI*|0 < MI < 1) = 0.3529					

*, **, and *** imply the p-value is significant at 10, 5, and 1% significance respectively.

MI = mechanization index; SDI=Simpson Diversification Index.

Household characteristics such as sex of the head, educational status, experience in farming, family labor, and social capital are statistically significant variables affecting farm mechanization level. Variables that determine access to institutions like location of household (i.e., zone of the household), access to all-weather roads and distance to farm mechanization service centers are also statistically significant enough in affecting the farm mechanization. Variables that indicate households’ economic activities, types and endowments of resources like participation in markets and off-farm activities, land size (ha), land fragmentation and livestock size (TLU) are also statistically significant in determining the level of farm mechanization.

Accordingly, the result of Tobit model (Table 6) shows that male-headed households are significantly less likely to use farm mechanization. It was hypothesized that female-headed households are resource-poor and have low power on resources and hence, deprived of technology adoption [47,48]. However, opposite to the hypothesis, being female-headed household increases the level of farm mechanization by 10.59% while it increases the level of farm mechanization by 0.0920 and 0.0322 for the whole sample and for those who already started mechanized farming, respectively ceteris paribus. Hence, being a female-headed household significantly affects the adoption of farm mechanization positively. Previous studies done like [27,34] found that women were less knowledgeable about the effects and advantages of mechanization due to some factors such as less formal education, an inability to attend extension services, and limitations of women’s movements outside the household and hence, they were low adopters. However, females may tend to use farm mechanization technologies to overcome family labor shortage and working animals like oxen, as it is the case indicated under descriptive analysis result where female headed households have a smaller number of oxen family labor (Table 5). As females are resource poor and possess less livestock (oxen) and low family labor, farm mechanization could be the only way-out for survival in this area.

Experience in farming significantly and positively explained the level of mechanization at the 5% level. The result of marginal effects depicted that if experience in farming increases by one year the level of farm mechanization (index) increases by around 0.26%, keeping other variables constant. It also revealed that increasing farm experience by a year will increase the level of farm mechanization in the whole population under study and for those already started using farm mechanization technologies by 0.0022 and 0.0013 respectively. Similar studies also showed that age (proxy for farming experience), has positively affected the use of tractor powered machineries in African countries like Kenya, Niger, Zambia, and Zimbabwe and also for the pooled sample in general [27] significantly [28]. Also found that age has positive effects on farm mechanization service purchase of rice farmer in China. However [29], found that the elderly farmers are less likely to adopt harvesting machines may be due to nonrealization of direct impacts on the yield and net profit of the machine.

Households’ head and other family members (especially spouse) educational status is an important factor in enhancing farm technology adoption. It is believed that education, whether formal or informal, will improve awareness, knowledge and skill of a farmer on the importance and how to use of the farm machineries [29]. The result of this finding also showed that household head educational status affects farm mechanization level positively at the 5% level of significance. A unit change in educational status will change level of farm mechanization by around 0.70%, whereas it increases the level of farm mechanization by 0.0056 and 0.0033 for whole population domain and for those who are already using farm mechanization technologies, respectively. According to study in some African countries including Ethiopia and South Asia, the household head education level is statistically significant in explaining the use of tractor-powered mechanization [27,29,30]. Similarly [31], also found that an increase in household heads’ education increased the probability of adoption of mechanization practices.

Spatial location of household (zone) is negatively affecting the level of farm mechanization and is significant at the 10% level of significance. According to the marginal effects result, farms in West Arsi are more mechanized and the model output indicated that shifting a household’s location from West Arsi to Arsi will reduce levels of farm mechanization by around 5%. Similarly, being in Arsi zone can reduce the farm mechanization level for the whole population and for those already practicing the technologies by 0.0419 and 0.0247, respectively. This could be due to the topography of the farmland, which is plain especially around the Gedeb Asasa district and convenient for mechanization and number of federal and regional state farms in and nearby districts like Ardayta agricultural, technical and vocational college, Oromia seed enterprise and Gofer farms. The presence of these farm organizations can have a positive impact in mechanizing private smallholder farm by providing machinery rental service and promoting mechanization. Other also reported that farm mechanization technologies ownership and adoption are affected by spatial location [[32], [33], [34]].

The household’s residence distance to farm mechanization service providing center is statistically significant at the 1% level of significance and is negatively affecting the level of farm mechanization. The marginal effect coefficient revealed that if the center of mechanization service provider is far from a household residence by a kilometer, the probability of using farm mechanization will be reduced by about 0.94% while the level of farm mechanization will be reduced by 0.0079 for population understudy and by 0.0047 for technology users.

The household’s wealth indicators like landholding and size of livestock are the most important significant variables in determining the level of farm mechanization. Accordingly, total land cultivated by a household (hectare) positively affects the level of farm mechanization significantly at the 5% level ceteris paribus. According to the result, as cultivated land size increased by 1 ha, the probability of using farm mechanization can be increased by 2.70% while the level of farm mechanization of the whole population domain and those started using farm mechanization will increase 0.0227 and 0.0134, respectively. Similar studies by Ref. [27] in Ethiopia and somewhere in African countries showed that probability of using animal-powered mechanization increases with landholding size. Similarly, farm size significantly increases the probability of using tractor-powered mechanization in Egypt, Niger, Zambia and Zimbabwe [27] and somewhere else [25,35,36].

Livestock possession measured in Tropical Livestock Unit (TLU) is another variable that explained the level of farm mechanization significantly at 5% negatively which is due to the fact that livestock are major sources of agricultural working power and mechanization technologies are substitutes for animal power. This finding is also consistent with other findings somewhere in the world where an increase in the level of farm mechanization significantly decreasing the use of draft animal power [49]. The marginal effect results showed that increasing the size of livestock holding by one TLU would decrease the probability of using farm mechanization by 0.80%, whereas it reduces the level of farm mechanization by 0.0067 and 0.0040 for the whole study population and for those who have already started using farm mechanization, respectively.

Land fragmentation measured by the number of farm plots possessed is another variable that affects farm mechanization level negatively at the 1% level of significance. This result also agrees with the findings of [[23], [24], [25], [26]]. Result of marginal effects shows that the increase in the number of farm plots by one unit will reduce the probability of using farm mechanization by 5% while it decreases the level of farm mechanization by 0.0421 and 0.0249 for the whole of the population under consideration and for those who started using farm mechanization technologies, respectively.

Total family labor in man-equivalence is negatively affecting farm mechanization level and significant at the 1% level. This result is in line with the assumption that labor availability in a household will reduce levels of farm mechanization due to the substitution nature of the two inputs, and it is similar to others findings [27,37,38]. The marginal effects result revealed that an increase of family labor by one unit decreases the probability of using farm mechanization by around 0.60%, whereas it reduces the level of farm mechanization by 0.0049 and 0.0029 for the population in the study area and current users of the technologies respectively.

Another important finding from this study was the positive and significant relationship between crop output market participation and the level of farm mechanization among farming households which is statistically significant at a 1% level. Our finding is also similar to others finding [39]. According to the findings, participation in crop output market can increase households’ probability of farm mechanization use by around 9%. The marginal effects further showed that increasing market participation by a unit can increase the level of farm mechanization by 0.0747 and 0.0593 for population of study and current users respectively.

There is multidimensional concept of social capital [50,51]. Accordingly, index of social capital is constructed from seven dimensions of social capital. Social capital is crucial for technology adoption and wellbeing as it is supposed to improve social networks and access to information; Hence, it is expected to influence technology adoption positively. However, in our finding a membership to different village institutions are negatively affecting the level of farm mechanization at 5% significance level. This is because having stronger social capital and bond enables households to get access to pooled labor through other means like debo, and wonfel and can access working animals (oxen and horses) easily and this result is consistent with the finding of [51]. The result of marginal effects showed that the increase in index of social capital decreases the decision to use farm mechanization by around 13%, whereas, it decreases the level of farm mechanization by 0.1099 and 0.0649 for the whole population understudy and for those already using the technologies respectively.

Access to all-weather road is also one of the variables that affect probability of using farm mechanization and the level of farm mechanization at the 5% level of significance. The finding of [34] also supports our findings. The marginal effects show that the probability of farm mechanization users who have access to all-weather roads increases by 7.07% as compared to those who do not have access while the level of farm mechanization increases of 0.0581 and 0.0452 for the whole population and for users of farm machineries respectively.

Participation of households’ member in off-farm economic activities is significant at 10% in explaining the level of farm mechanization. It increases, ceteris paribus, the probability of using farm mechanization by 4.40% while the result of marginal effects further revealed that the increase in off-farm activity participation will increase the level of farm mechanization by 0.0373 and 0.0190 for whole population and current user sample households respectively. Similar findings were reported by Refs. [27,32]. As per the assumption, use of farm mechanization of a household can save more time to participate in more nonfarm activities while the participation will in turn generate additional income to invest in farm mechanization technologies and they are supplementing each other.

## 4 Conclusion and recommendation

### 4.1 Conclusion

In general, use of farm mechanization in the study area is widely common. Two-wheel tractors, engine driven stationary threshers, four-wheel tractors and combine harvesters are the most important mechanization machineries. But the result showed that heavy duty machineries, namely 4-wheel tractors and combine harvesters are the most widely used among others where each of them is used by around 86% and 78% households respectively. The small horse power 2-wheel tractors are not appropriate due to the nature of the soil which is heavy to plow. Generally, at least around 88% of the households are using either of the mechanization technologies. Wheat and barley are the two major crops that cover around 81% of total cultivated land and are the more mechanized crops. Hence, the area is cereal dominated and can be said also wheat-barley belt area. The mean mechanization level of study area is 35% while the mechanization index for wheat and barley are 38.46% and 34.89% respectively, with a mean value of 38.46%. The mean land size of the sum of the two barley and wheat crops is 1.39ha, while mean landholding per household is 1.88ha. Mean wheat farm size and barley farm size are 0.86ha and 0.53ha respectively. Mean wheat farm size and barley farm size are 0.86ha and 0.53ha respectively.

Econometric model’s output shows that sex, educational background, experience in farming, family labor availability and social capital are statistically significant variables in influencing levels of the farm mechanization index. Variables that determine access to institutions like location of household (i.e., zone of the household), access to all-weather roads and distance to farm mechanization service provider centers are statistically significant. Variables that indicate households’ economic activities, types and endowments of resources like participation in markets and off-farm activities, cultivated land size (ha), land fragmentation and livestock ownership (TLU) are also statistically significant in determining the level of farm mechanization. Land topography which is proxied by administrative zones was also significant variation implying West Arsi is the more mechanized area as a location (zone) was turned out to be significant. Similarly, highland districts of the two districts are less mechanized compared to mid-highland areas citrus paribus. In general, high animal feed-cost, labor shortage, the higher wage rate for hire agricultural laborer and demand for a better life to reduce work drudgery are the most important driving factors towards farm mechanization in the study area.

### 4.2 Recommendations

Based on the findings of this study, the following recommendations are made for both policy makers and development practitioners to increase the level of farm mechanization.

1. **Land clustering, introduction of land augmenting mechanization technologies and consolidation:** In order to enhance farm mechanization level, the recent government initiatives cluster farming shall be scaled-out. Because the most important variable that positively affected the level of farm mechanization is farm size and number of farm plots (i.e., a measure of land fragmentation), and that improves the farm size under machineries operations. Per capita landholding is decreasing from time to time due to population pressure as farm land is continuously divided among descendants due to sub-division at inheritance. Hence, land augmenting mechanization technologies such as irrigation technologies and appropriate technologies for small-scale farms shall be introduced continuously. Households possessing larger cultivated land and less fragmented (smaller number of farm plots) have higher levels of farm mechanization. This implies that, to improve farm mechanization level and modernize Ethiopian agriculture, though it could be difficult in short-terms, consolidating farms and improving farm holding size (economizing farmland size) can help are important factors.
2. **Credit Facilitation for custom service providers:** In countries like Ethiopia where agriculture is dominated by small-scale and subsistence, individual-level machineries ownership could be almost impossible. For instance, none of surveyed farmers have any of farm mechanization machineries and our survey result evidenced that the growth of outsourcing services has enabled farms in the study area to mechanize irrespective of machine ownership. Hence, the best approach to mechanize agriculture is through rental service provision. From the KII and focus group discussion with service provider machinery owners, the newly launched credit facilities by commercial banks like Oromia Cooperative Bank and Development Bank of Ethiopia are contributing much in this regard. Machinery lease financing credit scheme by the Development Bank of Ethiopia is also contributing a lot in availing machineries for service rental provisions. Hence, continuing this credit system of machinery lease financing and availing another credit system can also enhance farm mechanization.
3. **Establishing nearby custom service centers**: Results revealed that household’s zonal location, access to all-weather road and distance from farm mechanization service centers are significant variables in determining the level of farm mechanization. Accordingly, households located in West Arsi and near the service centers are more mechanized. Moreover, transportation of large machineries, especially combine harvester, is highly affected by road access and topography. Secondary data also revealed that farm machineries are more available in West Arsi than in the Arsi zone due to availability of state farms, Oromia seed enterprises and more convenient land topography that attracts more investors, among others. Hence, availing farm mechanization service centers either by government or creating capacity of private service providers at appropriate and accessible location can enhance farm mechanization level. Similarly, full-filling infrastructure facilities mainly rural road can enhance farm mechanization level in the study area.
4. **Education and training of farmers**: Knowledge and skill can play significant role in determining adoption of farm mechanization. Awareness creation and knowledge about the importance of farm mechanization technologies and skills on how to use farm mechanization can improve and change farmers' attitude towards technology adoption. The result of econometric model showed that educated farmers have higher levels of farm mechanization. Hence, expansion of formal and informal (adult) education is important to enhance the level of farm mechanization.

## Author contribution statement

Tamrat Gebiso - conceived and designed the analysis, interpreted the data, and wrote the paper.

Mengistu Ketema-contributed analysis tools.

Arega Shumetie-contributed analysis tools.

Getachew Legese-contributed analysis tools.

## Data availability statement

Data will be made available on request.

## Declaration of competing interest

The authors declare that they have no known competing financial interests or personal relationships that could have appeared to influence the work reported in this paper.
