# Supplementary material for: Determinants of farm mechanization in central and southeast oromia region, Ethiopia
Source: Heliyon. 2023 Jul 17;9(7):e18390. doi: 10.1016/j.heliyon.2023.e18390 (PMC10375850; doi:10.1016/j.heliyon.2023.e18390)
Supplement: Multimedia component 1 [file mmc1.pdf]

Farm Mechanization, Commercialization, and Crop Diversification in central and Southern Oromia Region, Ethiopia

Ph.D. Dissertation Research Survey Questionnaire
Postgraduate Program Directorate, Haramaya University

General Instruction for Enumerators

1. Please introduce yourself before starting the interview according to local customs.
2. Inform the purpose of the study, which is to collect first-hand data that will be used only for research activity writing on the above-indicated topic only for knowledge purposes.
3. Please be careful with the estimates when exact measurements are not available.
4. Carefully read and follow the instructions while you are starting the interview.
5. Do not forget to thank when you finish your interview

Household ID

Date ____________________

Questionnaire ID (001-385): ________________________

Administrative zone code: ______________; District code _________ *Kebele code* _________

Enumerator’s name (code): ___________ Signature: _____________

Thesis the objectives

In general, this study will focus on farm mechanization of major crop, commercialization, and crop diversification and their inter-linkages in the central and southeastern parts of the Oromia regional state while the specific objectives are:

1) To estimate the farm mechanization level and identify its determinants for major crops
2) To estimate the level of economic efficiency of farming households
3) To estimate the impacts of farm mechanization on farm labor employment, economic efficiency, household income, and intensity of farm inputs used for major crops of the area;
4) To identify determinants of farm commercialization;
5) To estimate crop diversification level and identify determinants of crop diversification; and

To analyze the inter-linkage among farm mechanization, commercialization, and crop diversification in the study area

Household ID

Date ____________________

Questionnaire ID: ________________________ Region _________________

Administrative zone code: ______________; District code _________ *Kebele code* _________
Enumerator's name (code): ___________ Signature: _____________

Part I: Household Characteristics

1. Name of the respondent (Household head) ______________________________

2. Sex of the household head: 1. Male 0. Female

3. Age of respondent _____________ years

4. Marital status:

1) Single 2) Married 3) Divorced 4) Widow

5. Educational status of household head/respondent: 1. Illiterate 2. Read and write

3. Informal (religious school)

6. If the household head is literate what is his/her years of schooling? ________

7. Family size of the household

S. N.	Age category	Number of males	Number of females
1	Bellow 10 years		
2	10-14 years		
3	14-16 years		
4	17-64 years		
5	Above 64 yrs		

8. What is the number of family members working full-time in the farm? ____________

9. What is your experience in farming (years): ___________________ years

Part II: Access to Services

1. Distance to infrastructures/service centers:

No.	Infrastructure/service center	Distance (Km) from residence
1	Nearest market	
2	Farmer cooperative center	
3	Main road	
4	FTC	
5	Main market	
5	Mechanization service providing center	
6	Major (district's) town	
7	DA office	

2. Do you have an all-weather road near your residence? 1) Yes 0) No

3. Do you have contact with DA? 1) Yes 0) No

4. If 'Yes', how often do you contact DA per month? ________________ times per month.

5. Do you have access to credit (can you borrow if you need it)? 1) Yes 0) No

6. If yes, for what purposes did you borrow money for 2012/13 or 2013/14 production seasons?

No.	Purpose of credit	Amount	Source
1	Fertilizer		Relatives/friends
2	Seed		Ekub
3	Agro-chemicals purchase		Saving and credit associations
4	To participate on off/non-farm activities [it includes fattening, petty trading etc.]		Banks
5	To fulfill basic needs (food, clothes)		Cooperatives/unions
6	Children's school		
7	To pay for/buy farm mechanization technologies services		

7. Are you a member of any of these associations in the last two years? (list them)

Type of associations **(Use code A)**	For how many years?	Role in the institution? **(Use code B)**	Still a member now? **(Use code C)**

**Code A**	**Code B**	**Code C**
1. Input Supply/service coops /union	1. Elected official	1. Yes
2. Crop producer and marketing coops	2. Ordinary member	0. No
3. Local administration (*Kebele*)	3. Agricultural cadre	
4. Women's Association	4. Model farmer	
5. Youth Association	5. Cashier	
6. Mosque/Church Association	6. Coordinator	
7. Saving and credit group	7. Secretary	
8. Funeral Association (*Idir*)	8. Militia	
9. Seed producers' cooperatives	9. Store keeper	
10. Water User's Association	10. Executive committee member	

8. Main roofing material of your main residential house? **1. Grass thatched** **2. Iron sheet**

Part II: Household Land Holding and Land Use Pattern

1. Land possession during the 2013/14 E.C cropping season

**No.**	**Landholding**	Size/area in ha	Value of land in year basis*
1	Total cultivated land		
2	Total own landholding (from PA)		
3	Grazing land		
4	Homestead land		
7	Land rented in		
8	Land shared in		
9	Land rented out		
10	Land shared out		
11	Total land		

**Take rental value since it is the best proxy for price of land*

2. What is the number of plots/parcels of your agricultural land? _______________.

3. What is the size of each plot?

Plot number	Plot size (ha)
1	
2	
3	
4	

4. In general what is the fertility status of your farmlands?

1) Good 2) Medium 3) Poor

Part III: Off/non-farm activities

1. Do you or/any member of your family have an off-farm/non-farm job? 1. Yes 0. No

2. If yes, indicate the type of off/non-farm activities and annual income for the year 2013/14

S/N	Off/non-farm activities	1. Yes<br>0. No	Payment mode<br>1. In kind 2. Cash	Total annual income(birr/annual)
1	Livestock trading			
2	Crop trading (grain, vegetable …)			
3	Petty trade (consumer goods)			
4	Hired laborer (on/non-farm activities)			
5	Rental income (land, house...)			
6	Income from aid (safety net)			
7	Salaried employment			
8	Remittance			

Part IV: Crop Production, marketing, and Crop Diversification

1. Crop production and income from crops in 2013/14 cropping season

Crop types grown	Cultivated Area (ha)	T. produced (kg)	The quantity consumed kg)	Quantity saved for seed(kg)	Did you sell? 1. Yes 0. No	Quantity sold (kg)	Selling price (birr/kg)
Wheat							
Barley							
Maize							
Sorghum							
Teff							
Common bean							
Field pea							
Chick peas							
Faba bean							
Khat							
Potato							
Sweet potato							
Onion							
Tomato							
Hot pepper							
Garlic							
Carrot							
Mango							
Avocado							
Papaya							
Banana							
Sugar cane							
Others:-							

2. In general, the sale of your crop is distressed or surplus? 1. Distressed 2. Surplus

3. Your production goal or target is: 1. Totally for household consumption
   2. Mainly for consumption but to some extent for market 3. Equally for both consumption and market 4. Mainly for market purposes but for consumption to some extent 5. Entirely for market purpose

4. Crop produced mainly for market purposes is 1. Cereals 2. Pulses 3. Vegetables

5. Your Production goal is market-oriented; do you agree? 1. Strongly disagree
   2. Disagree 3. Neither disagree nor agree 4. Agree 5. Strongly agree

6. Do you store your agricultural product for later sale? 1. Yes 0. No.

7. If you store your product, for how many months do you store it? _____________ months.

8. What criteria do you consider while selecting a crop for production? (Rank them 1 to 7)

No.	Selection criteria	Rank	No.	Selection criteria	Rank
1	food types accustomed to the family/society		5	Suitability of the crop for rotation	
2	Suitability of the crop to my land		6	Availability of inputs	
3	Availability of market/good price		7	Availability of its mechanization technologies	
4	Availability of land				

9. If you do not store your product for later sale, why?

1. I do not have enough (surplus) products for sale
2. I do not have conducive (appropriate) and safe storage facilities
3. I do not have enough space for storage
4. Because I need money for immediate obligations (purposes)
5. I do not see the importance of storing products for future sale

10. What are the two more important constraints in marketing your agricultural products (crops)?

1. Low demand for products 2. Price fluctuation
3. Middlemen's problems 4. Absence of market information
5. Absence of road access 6. The absence of transportation means

11. To whom do you sell your agricultural products?

1. Directly to rural consumers 2. Directly to urban consumers
3. To retailers and assemblers 4. To wholesalers
5. Agricultural cooperatives

12. Do you have mobile phone? 1. Yes 0. No

13. Do you have equines to transport your agricultural products to market? 1. Yes 0. No

14. What means of transportation do you use to transport your agricultural products to market?

1. Equines back 2. Cart 3. Vehicle 4. Human back

15. Do you get market Information (like price) for your agricultural products? 1. Yes 0. No

16. List two crops that you mainly grow for market purposes: first ______; Second ________.

18. Do you have a contract farming agreement for the marketing of any of your crops?

1. Yes 0. No

19. If you do have a contract farming agreement, for which crop? ____________________.

20. If you do have a contract farming agreement, who is the contracting party? ______________.

21. In general do you use the improved seed for your major crops regularly? Yes, 0. No

22. Is your crop production diversified or specialized? 1. Specialized 2. Diversified

23. If it is diversified, why? 1. To mitigate the risk of crop failure 2. To mitigate the risk of price fluctuation 3. To get food self-sufficiency 4. To diversify labor pressure during peak time

24. What is (are) the main reason for not to diversify your crop production? (Rank them)

No.	Reason for not to diversify	Rank
1	I need crops that are more commercialized	
2	I need more mechanized crops	
3	My land size is too small and limits my diversification needs	
4	Inputs like improved seed availability restrict my need	

25. Which crop types cover most parts of your land? 1st crop ______=_________%; 2nd crop _______= ________%; 3rd crop ________ = ______% and 4th crop ______ = _______%.

Part V: Agricultural Mechanization-Related Issues

1. Did you use any farm mechanization technologies (listed in Q.2 below) in the (2022) 2013/4 E.C. Production season? 1. Yes 0. No

2. Which Farm mechanization are you using currently (this year)?

No.	Farm Mechanization type	Response	Supplier*
1	Two wheels tractors	1. Yes 0. No	
2	Four wheels tractors	1. Yes 0. No	
3	Walking behind harvesters	1. Yes 0. No	
4	Engine-driven threshers	1. Yes 0. No	
5	Combine harvesters	1. Yes 0. No	

*1. Own 2. Cooperatives/Union, 3. Government enterprises; 4. Private owners

3. How often do you use this farm machinery? 1. Rarely 2. Sometimes 3. Every year (regularly)

4. What are the constraints you face in using farm mechanization in general? (Rank 1-4)

No.	Constraints to use farm mechanization technologies	Rank
1	Unavailability of the technologies in the area	
2	High price of the service or technologies to purchase	
3	Technical incapability on how to use the technology	
4	Ignorance to the technology	

5. Do you participate in training on farm mechanization issues? 1. Yes 0. No

6. Do you participate in demonstrations of farm mechanization? 1. Yes 0. No

7. Do you participate in field days of farm mechanization? 1. Yes 0. No
8. Do you get extension services on farm mechanization? 1. Yes 0. No
8. If your answer is yes, how do you rate extension service provision on farm mechanization?
 1. Poor 2. Neither poor nor good (medium) 3. Good
9. Properties of your farm plot 1. Slopy/rocky and inconvenient for mechanization
 2. It is neither too sloppy nor rocky to use farm mechanization technologies

Part VI: Livestock ownership and annual income from livestock

No.	Type of livestock	Current owned	Sold within a year since September	Sales amount (ETB)
1	Oxen			
2	Cow			
3	Calf			
4	Weaned calf			
5	Heifer			
6	Horse			
7	Mule			
8	Donkey (adult)			
9	Donkey (young)			
10	Sheep (adult)			
11	Sheep (young)			
12	Goat (adult)			
13	Goat (young)			
14	Chicken /poultry birds			
15	Beehives		Bee swarm	
			Honey (kg)	
